# Longitudinal *In Vivo* SPECT/CT Imaging Reveals Morphological Changes and Cardiopulmonary Apoptosis in a Rodent Model of Pulmonary Arterial Hypertension

**DOI:** 10.1371/journal.pone.0040910

**Published:** 2012-07-17

**Authors:** Michael L. Paffett, Jacob Hesterman, Gabriel Candelaria, Selita Lucas, Tamara Anderson, Daniel Irwin, Jack Hoppin, Jeffrey Norenberg, Matthew J. Campen

**Affiliations:** 1 Department of Pharmaceutical Sciences, University of New Mexico Health Sciences Center, Albuquerque, New Mexico, United States of America; 2 inviCRO, Boston, Massachusetts, United States of America; 3 Radiopharmaceutical Sciences Program, College of Pharmacy and Keck-UNM Small-Animal Imaging Resource, University of New Mexico Health Sciences Center, Albuquerque, New Mexico, United States of America; Vanderbilt University Medical Center, United States of America

## Abstract

Pulmonary arterial hypertension (PAH) has a complex pathogenesis involving both heart and lungs. Animal models can reflect aspects of the human pathology and provide insights into the development and underlying mechanisms of disease. Because of the variability of most animal models of PAH, serial *in vivo* measurements of cardiopulmonary function, morphology, and markers of pathology can enhance the value of such studies. Therefore, quantitative *in vivo* SPECT/CT imaging was performed to assess cardiac function, morphology and cardiac perfusion utilizing ^201^Thallium (^201^Tl) in control and monocrotaline-treated rats. In addition, lung and heart apoptosis was examined with ^99m^Tc-Annexin V (^99m^Tc-Annexin) in these cohorts. Following baseline imaging, rats were injected with saline or monocrotaline (50 mg/kg, i.p.) and imaged weekly for 6 weeks. To assess a therapeutic response in an established pulmonary hypertensive state, a cohort of rats received resveratrol in drinking water (3 mg/kg/day) on days 28–42 post-monocrotaline injection to monitor regression of cardiopulmonary apoptosis. PAH in monocrotaline-treated rats was verified by conventional hemodynamic techniques on day 42 (right ventricular systolic pressure (RSVP) = 66.2 mmHg in monocrotaline vs 28.8 mmHg in controls) and in terms of right ventricular hypertrophy (RV/LVS = 0.70 in monocrotaline vs 0.32 in controls). Resveratrol partially reversed both RVSP (41.4 mmHg) and RV/LVS (0.46), as well as lung edema and RV contractility *+dP/dt_max_*. Serial ^99m^Tc-Annexin V imaging showed clear increases in pulmonary and cardiac apoptosis when compared to baseline, which regressed following resveratrol treatment. Monocrotaline induced modest changes in whole-heart perfusion as assessed by ^201^TI imaging and cardiac morphological changes consistent with septal deviation and enlarged RV. This study demonstrates the utility of functional *in vivo* SPECT/CT imaging in rodent models of PAH and further confirms the efficacy of resveratrol in reversing established monocrotaline-induced PAH presumably by attenuation of cardiopulmonary apoptosis.

## Introduction

Idiopathic pulmonary artery hypertension (PAH) is a devastating lung vascular disease mainly afflicting women in their fourth decade of life [1]. PAH is associated with an extremely poor prognosis, with an estimated median survival of 2.8 years from time of diagnosis to death [2]. Despite significant advancements in disease management, there is no known cure for PAH and treatment regimens remain palliative. The pathobiology of PAH is characterized as an obliterative vascular disease of the small pulmonary arteries in which excessive proliferation of pulmonary artery endothelial and smooth muscle cells lead to remodeling of pulmonary arteries, increased pulmonary vascular resistance, and eventually right heart failure. Furthermore, right ventricular failure is the major cause of mortality in patients diagnosed with idiopathic or severe secondary varieties of PAH [3].

Because the disease is difficult to diagnose until later stages, much of what is known about PAH comes from animal models. Numerous models exist, with varying coherence to the human disease. Such models provide an opportunity to observe factors that may contribute to the early stages of pulmonary arterial remodeling, lesion formation, and right ventricular hypertrophy, as in humans we are mostly knowledgeable regarding outcomes of fulminant disease. Thus, a key advantage of animal disease models coupled with *in vivo* imaging techniques is the unique capacity to conduct serial, non-invasive studies that allow for a better understanding of disease progression and response to therapeutic intervention. Recent findings by Marsboom et al. [4] utilize ^18^F-fluorodeoxyglucose positron emission tomography in both monocrotaline and Sugen models of PAH to monitor the shift toward glycolytic metabolism lung vascular cells as well as therapeutic responses to dichloroacetate or imatinib. In a different study, therapeutic efficacy of the angiotensin II receptor antagonist varlsartan has been demonstrated by serial *in vivo* measurements of cardiac apoptosis utilizing ^99m^Tc-Annexin-V (^99m^Tc-Annexin) scintigraphy in the monocrotaline model of PAH [5]. With respect to conventional physiological assessment of classical indices of PAH, we established a time-course reflecting the development of increasing RSVP and right ventricular hypertrophy in the monocrotaline model [6]. Moreover, we found that oral administration of resveratrol attenuates these pathologic variables after the development of monocrotaline-induced PAH. Although controversial, allosteric activation of SIRT1 by resveratrol may be one of several protective mechanisms in addition to its anti-inflammatory and anti-oxidant related mechanisms [7]. In terms of cardiac function, resveratrol has been shown to improve contractility, diastolic function and attenuate cardiomyocytes apoptosis in a number of experimental models of cardiac dysfunction [8], [9], [10]. Furthermore, resveratrol improves survival in rodent models of heart failure and myocardial infarction by modulating cardiomyocyte energetic phenotype [11] and reversing left ventricular hypertrophy [12].

The present study explores a SPECT/CT characterization of the major pathophysiological changes in response to monocrotaline, a toxin selectively injurious to the pulmonary vascular endothelium that leads to pulmonary artery and right ventricular remodeling. Quantitative *in vivo* ECG-gated ^201^TI SPECT/CT imaging was performed to assess longitudinal changes in cardiac function, morphology and perfusion. Additionally, cardiac and pulmonary vascular apoptosis was assessed by ^99m^Tc-Annexin in control and monocrotaline-treated rats. Furthermore, parallel longitudinal studies examined the effects of chronic oral resveratrol administration on monocrotaline-induced cardiac and pulmonary vascular apoptosis by ^99m^Tc-Annexin radiographic imaging. Findings from the study highlight the value of longitudinal analysis in observing monocrotaline-induced RV dysfunction and the efficacy of chronic oral resveratrol administration on pulmonary as well as cardiac apoptosis.

## Results

**Figure 1:**
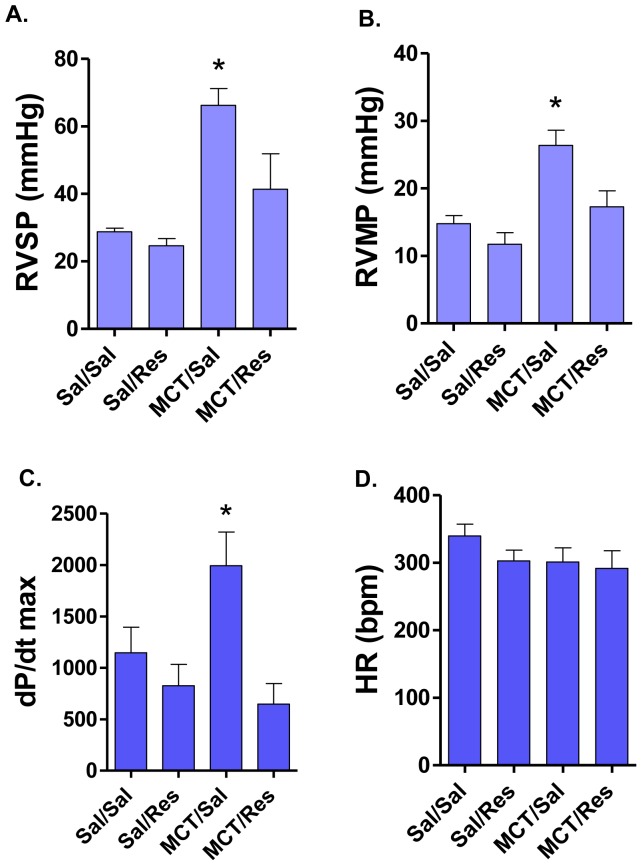
Monocrotaline induced significant changes in right ventricular systolic (A) and mean (B) pressure, which were alleviated by late treatment with resveratrol in the drinking water. Right-sided cardiac contractility, as assessed by +*d*P/*d*t_max_ from the RV pressure wave, was significantly elevated in the MCT-treated rats, and again reduced to control levels by resveratrol (C). No heart rate (HR) differences among the groups were noted, confirming a consistent level of anesthetic depth between groups during hemodynamic assessment (D). Asterisks indicate significant (P<0.05) elevation over other groups by ANOVA with Newman-Keuls Posthoc Comparison Test.

Similar to our previous report [6], a moderate monocrotaline dose (50 mg/kg) induced significant increases in peak and mean right ventricular pressure (Fig. 1A-B) when compared to saline injected rats. No effects of oral resveratrol administration (day 28–42) were seen in saline injected controls. However, resveratrol led to a significant attenuation in systolic and mean right ventricular pressures in monocrotaline injected rats which was consistent with our previous findings [6]. Interestingly, +*d*P/*d*t_max_ was elevated in the monocrotaline-saline group compared to controls (saline-saline) and resveratrol normalized the magnitude of +*d*P/*d*t_max_ to the level of controls (Fig. 1C). In an effort to determine whether anesthesia had a disproportional impact on these hemodynamic measures, heart rates were calculated and were found to be consistent between groups indicating a consistent level of anesthesia (Fig. 1D).

**Figure 2:**
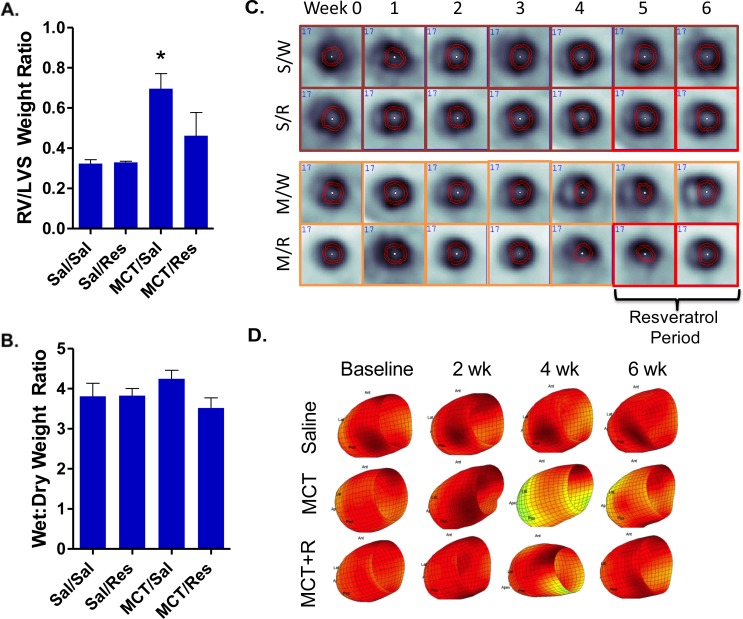
Right ventricular remodeling following MCT injection was evident from the ratio of right ventricular mass to left ventricular plus septal mass (RV/LVS); this effect was diminished by resveratrol treatment (A). No obvious effect on lung remodeling or edema formation was noted at 42 days post-MCT injection (B). Right ventricular enlargement was evident at 3–4 weeks in MCT/water-treated rats (M/W), with some resolution in MCT/resveratrol rats (M/R) from SPECT images of ^201^Thallium (C). No apparent changes were noted in saline/water (S/W) or saline/resveratrol (S/R) control rats. Notably, septal indentation, a result of increased right ventricular pressure, was evident as early as 2 weeks post-MCT (D). Asterisks indicate significant (P<0.05) elevation over other groups by ANOVA with Newman-Keuls Posthoc Comparison Test.

In addition to blood pressure measurements, the changes in mass of the right ventricular and left ventricular plus septa were examined across these cohorts. As expected, monocrotaline-induced PAH led to hypertrophy of the RV relative to the left ventricle plus septal wall (RV/LVS) as indicated in Figure 2A. Additionally, a slight increase in lung edema was noted (Fig. 2B), but was not significant in this moderate model of monocrotaline PAH. *In vivo* SPECT imaging revealed pronounced leftward septal wall deviation at week 4 in the monocrotaline-treated rats (Fig. 2C &D); however, radiologic analysis of RV morphology was limited to overt RV hypertrophy due to disproportionate ^201^Tl uptake in the left ventricle compared to the thinner walled RVs in the control rats. In a manner consistent with reduced RV systolic pressures (Fig. 1), resveratrol reversed both RV hypertrophy (Fig. 2A) and septal deviation (Fig. 2B & C) in monocrotaline-treated rats, demonstrating therapeutic efficacy.

**Figure 3:**
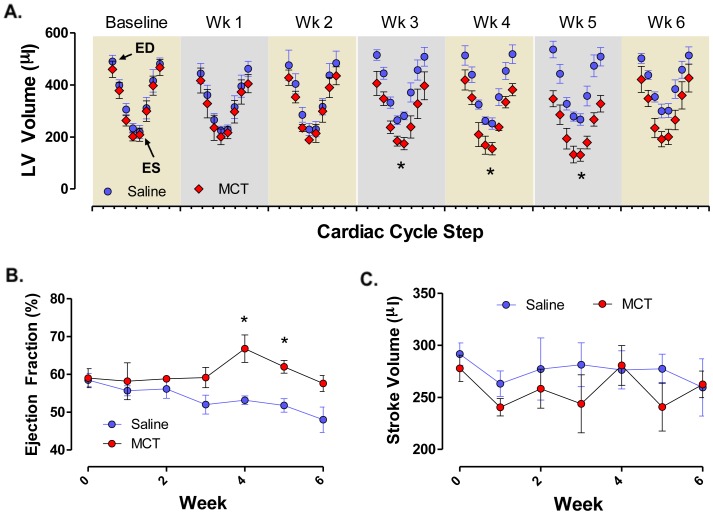
Left-sided cardiac cycle changes over the 6-week period of MCT-induced PAH pathogenesis (A). ECG-gating methods enabled the distribution of ^201^Thallium decay detections into 8 bins between electrocardiographic R waves. Cardiac cycles from end diastole (ED) to subsequent end diastole are shown, with end systole (ES) in the middle. Both treatment groups showed nearly identical left ventricular function at baseline and up until week 3 post-MCT injection. At week 3, and progressing through week 5, a significant reduction in the overall volume of the left ventricle was noted. This corresponded with increased ejection fraction (B) and no net change in stroke volume (C) over the same time period. Asterisks indicate significant (P<0.05) elevation over other groups by 2-way (time, treatment) ANOVA.

**Figure 4:**
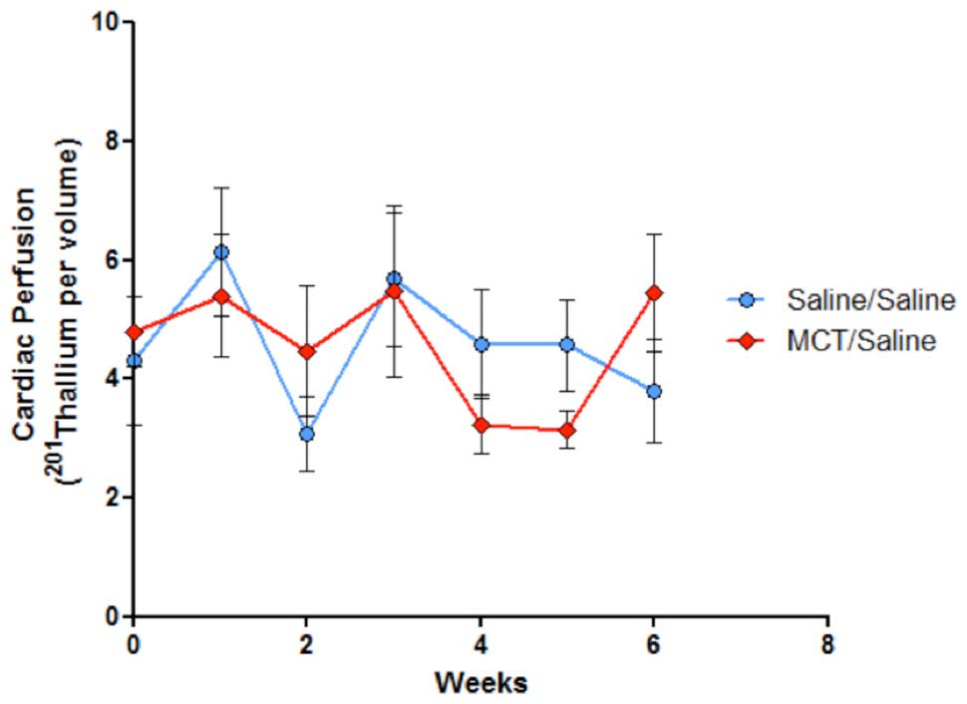
Global cardiac perfusion, as assessed by 201Thallium detection, showed no significant differences between groups or trends across the 42-day imaging period.

Given the overt changes on RV hypertrophy and cardiac wall morphology by monocrotaline, we set forth to examine left ventricle diastolic and systolic volumes from ECG-gated ^201^Tl SPECT images. Monocrotaline induced progressive reductions in LV residual volume, which is consistent with the notion of ventricular interdependence (Fig. 3A). As mentioned above, the SPECT image analysis software algorithm was unable to reliably compute RV diastolic and systolic volumes in control rats because of disproportional ^201^Tl signal from the LV, which consistently obscured RV ^201^Tl signal except in extreme cases of RV hypertrophy. Further *post hoc* analysis of ejection fraction (%) and stroke volume (µL) was derived from diastolic-systolic filling curves (Fig. 3B & C). Interestingly, ejection fraction was elevated in monocrotaline-treated rats at weeks 4–5 and then returned to baseline at week 6, whereas ejection fraction was unchanged in saline controls. Moreover, stroke volume was not changed in either group suggesting an increase in contractility. Therefore, we assessed RV contractility (*+dP/dt*_max_) and found a significant increase in *+dP/dt*_max_ 6 weeks following monocrotaline treatment compared to saline controls, which resolved in the monocrotaline-treated rats receiving resveratrol (Fig. 1C). In addition to cardiac function utilizing ECG-gated ^201^Tl imaging, we were able to longitudinally evaluate net cardiac perfusion (Fig. 4). No consistent changes in net cardiac perfusion were observed as a result of monocrotaline, although a slight non-significant trend occurred at week 5.

**Figure 5:**
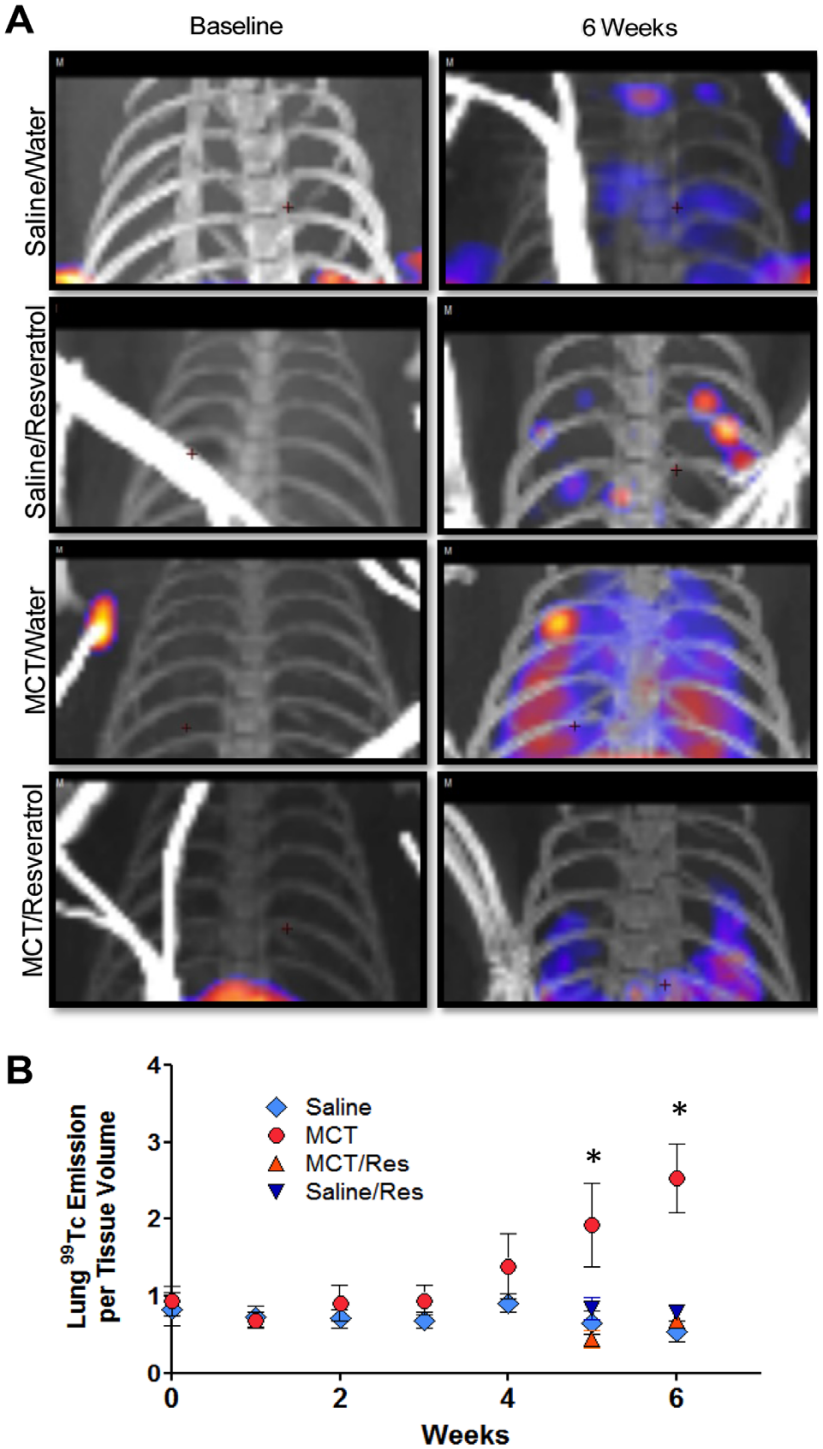
Representative SPECT/CT images of lung ^99^Tc-annexin at day 0 and day 42 from one subject per group. CT images include the ECG wires in various positions. Quantitative assessments of ^99^Tc energy from the lung region, with cardiac region subtracted out, are shown graphically, below. Asterisks indicate significant (P<0.05) elevation in the MCT-only group compared to other groups by 2-way (time, treatment) ANOVA.

**Figure 6:**
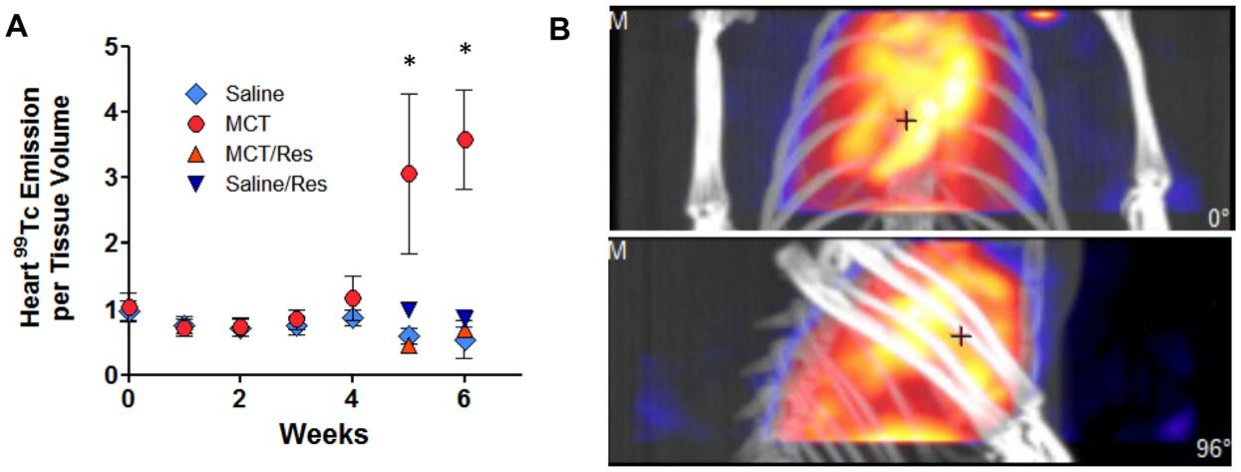
Quantitative assessments of ^99^Tc-annexin energy from the cardiac region, without consideration of pulmonary signals, are shown graphically. A severe induction of cardiac apoptosis was observed in one MCT-only rat, depicted from two positions in the figure to the right. Asterisks indicate significant (P<0.05) elevation in the MCT-only group compared to other groups by 2-way (time, treatment) ANOVA.

Longitudinal *in vivo* quantification of pulmonary and cardiac ^99m^Tc-Annexin SPECT imaging was performed in saline- and monocrotaline-treated rats receiving either tap water or resveratrol. Thoracic SPECT/CT images illustrate this increase in ^99m^Tc-Annexin binding in monocrotaline-treated rats and the sparing effect of resveratrol on lung apoptosis (Fig. 5A). Monocrotaline induced a progressive increase in ^99m^Tc-Annexin binding in the lung compared to saline controls (Figure 5B). This elevation in ^99m^Tc-Annexin began in the lungs of some monocrotaline-treated rats as early as 3 weeks post injection and was consistently elevated at weeks 5 and 6. Specifically, 2 premature lethalities were observed in the early onset of increased ^99m^Tc-Annexin binding *(data not shown)*. Similar to these pulmonary findings, monocrotaline induced a corresponding increase in ^99m^Tc-Annexin binding in the heart in which these elevations were only observed during weeks 5–6 post monocrotaline (Fig. 6A). Furthermore, resveratrol treatment abrogated this increase in ^99m^Tc-Annexin in monocrotaline-treated rats and had no measureable effect of altering apoptosis in saline control rats.

## Discussion

The present study confirms the ability of orally-administered resveratrol to provide therapeutic effects in an established model of pulmonary hypertension. The use of non-invasive, *in vivo* SPECT-CT imaging offered the ability to examine functional, morphological, and biomolecular changes longitudinally in this model, which revealed an important role of resveratrol in blocking the development of apoptosis in the hearts and lungs of monocrotaline-treated subjects.

*In vivo* SPECT/CT imaging revealed a number of pathophysiological changes in the cardiopulmonary system over a 6-week period of PAH development. Most prominent were changes in the diastole/systole filling curves (Fig. 2A), evidenced by a significant reduction in LV filling volumes during the cardiac cycle developed from weeks 3–5 in monocrotaline-treated rats. Furthermore, volumetric SPECT assessment of LV function corroborated the development of septal deviation, as evidenced by the reduced ventricular reserve volume and morphological findings, which nicely illustrates the concept of ventricular interdependence. Under normal physiological conditions, the right ventricular force generation and diastolic behavior is influenced by the left ventricle [13], whereas when RV pressure increases there is an impingement on left ventricular diastolic filling. Importantly, and consistent with what is found in compensated RV dysfunction, LV function was unimpaired in terms of overall output [14]. However, in scenarios where the right ventricular function is compromised, such as in Cor Pulmonale, secondary LV diastolic dysfunction is likely and the impairment of cardiac output is of clinical concern.

Interestingly, we observed an increase in ejection fraction without increases in stroke volume, which could potentially be explained in part by increases in contractility (illustrated by compensatory increases in *+dP/dt_max_*) along with the reduction in end-diastolic volumes. Although we did observe an increase in ejection fraction in monocrotaline-treated rats, this parameter appeared to resolve due to spontaneous disease regression or secondary to RV failure. Indeed, in a slightly more severe model of monocrotaline-induced PAH (using 60 mg/kg instead of 50 mg/kg), Correia-Pinto et al. [15] demonstrated substantial LV diastolic dysfunction and biomolecular changes at week 6 post-injection. Similar findings were also reported by Campian et al. [5], where echocardiographic assessment of RV failure was evident in monocrotaline-treated rats and was significantly delayed with long-term administration of valsartan, although losartan proved to have little therapeutic benefit in secondary human forms of PAH [16]. Aside from this caveat related to the discrepancy between the monocrotaline model and various forms of human PAH, this report documented the progression of RV dysfunction in rats. This was attributed to, in part, by an increase in early stage myocardial apoptosis and highlights the importance of *in vivo* longitudinal study design in order to better identify temporal events and how these markers relate to cardiac function during disease progression.

The plant-derived phytoalexin, resveratrol, has recently been shown to prevent [7] as well as reverse monocrotaline-induced PAH [6]. It has not clear if the principle therapeutic action resides within the pulmonary vasculature and/or the myocardium. The present study provides robust support for inhibition of cell death, both in the heart and lungs, as a major therapeutic outcome of resveratrol treatment, however we cannot be certain whether these effects are entirely the result of resveratrol; an oxidation product of resveratrol or metabolite of this poorly bioavailable phytoalexin. While Csiszar et al. [7] did not show significant increases in pulmonary endothelial apoptosis, this was possibly due to assessments of PAH endpoints at an earlier stage of disease. They examined tissues at 14 and 21 days post-monocrotaline, while we did not observe increased annexin signals until day 28 in some subjects, with consistently-elevated apoptosis at day 35 post-monocrotaline. Using ^99m^Tc-Annexin scintigraphy, Campian et al. [5] showed that the 60 mg/kg monocrotaline-treated model could induce right ventricular apoptosis as early as the third week post-injection and reaching a maximum at day 28, with confirmation by TUNEL staining and autoradiography. Vascular TUNEL staining has been shown to increase at day 28 in the monocrotaline-treated model [17] and other groups using different methods (i.e. Caspase-3 staining) have observed indications of vascular apoptosis at earlier time points [18]. In addition, Yang et al. [19] observed a histological improvement in right ventricular apoptosis by resveratrol at day 21 post-monocrotaline treatment although the resveratrol was administered prior to the development of PAH.

Taken together, these studies are consistent with regard to the onset of either cardiac or vascular apoptosis, despite discrepancies in monocrotaline dosing, and reinforce the utility of longitudinal *in vivo* studies designed to assess the efficacy of resveratrol to reverse established monocrotaline-induced PAH. In addition to the potential contribution of myocardial apoptosis to RV failure, increases in myocardial fibrosis also appear to influence disease progression in a number of heart failure models. Specifically, myocardial collagen deposition has been documented in a compensatory RV hypertrophy monocrotaline model [20] as well as overt failure (60 mg/kg) with the toxic pyrrole [5]. Although speculative, alternative therapeutic actions of resveratrol in the current study may be related to anti-inflammatory and/or anti-fibrotic effects described by Chan et al. [21], where this dietary compound decreased inflammatory cell infiltration, decreased cardiac fibrosis and improved both cardiac and vascular function.

One notable caveat is that the monocrotaline-treated model of PAH is associated with a substantial inflammatory response. Specifically, monocrotaline-enhanced IL-6 response and reduction in the clinically-relevant marker BMPR2 are known to contribute to the development of PAH [22]. In addition to these findings, Wang et al. [23] demonstrated monocrotaline-induced PAH is associated with differential perivascular T-helper activation. With respect to phosphatidylserine externalization as an early marker of apoptosis, activated macrophages also externalize these residues; thus, annexin may also serve as an inflammatory marker [24]. Resveratrol potentially mitigates components of the secondary inflammation caused by monocrotaline [7]. However, in the present study there was a 5-week delay in monocrotaline-induced ^99m^Tc-Annexin signal in the heart and lungs (Fig. 5 & 6) which was completely nullified with resveratrol. In previous work, we found that monocrotaline-associated inflammation was minimal at 6-weeks post-injection [6], thus the present findings strongly suggest that apoptosis is indicated by the annexin labeling.

Another distinct challenge with this imaging technology was the difficulty in assessing RV function and morphology in healthy animals. With a minor modification of the detection criteria, we were able to assess RV function in severe cases of PAH, but as these could not be compared with control animals, therefore we excluded these data. However, the potential exists to conduct efficacy studies on the reversal of RV dysfunction in models of established PAH. Finally, the monocrotaline-treatment model, while commonly used, has a number of limitations with regard to human disease. The major differences relate to the remodeling of the pulmonary arterioles, where in humans the development of plexiform lesions and myoendothelial transition can be observed, while monocrotaline causes hypertrophy of conduit and resistance vessels without the obliterative pathology in the capillaries. However, organ-level changes are quite consistent, especially with regard to right ventricular enlargement. Perhaps more importantly for the present application, imaging analysis of several related models, such as chronic hypoxia or hypoxia plus a VEGF inhibitor, would be readily conducted mirroring the present study design.

### Conclusion

Longitudinal *in vivo* SPECT/CT dual isotope imaging in a commonly used model of pulmonary hypertension revealed dynamic changes in cardiac function and cardiopulmonary apoptosis. The utility of this serial imaging technique could be applied to pharmacotherapy and efficacy assessment related to drug development across a broad range of experimental disease models.

## Materials and Methods

### Study Design

All procedures were approved by the University of New Mexico Office of Animal Care Compliance. Male Sprague-Dawley rats were obtained from a commercial vendor (Charles River) at approximately 300 g body weight. Rats were maintained in AAALAC-approved facilities with food and water available ad libitum.

Following a baseline imaging session, induction of pulmonary hypertension was achieved by a single intraperitoneal injection of monocrotaline at a concentration reported to cause modest disease (50 mg/kg, N = 12) (*6*), or an equivalent volume of sterile saline. At 28 days post monocrotaline injection, a cohort of rats (N = 12) was given resveratrol (Sigma Aldrich) in the drinking water (3 mg/kg/day). Resveratrol was administered via non-translucent water bottles and dosing was determined by average daily water intake which continued until the end of the study at Day 42. Fresh water containing resveratrol was changed every day to avoid problems with stability and solubility.

### Radiolabeling and Incorporation Yield

Thallium-201 chloride, USP, a sterile, isotonic, aqueous injectate, pH = 4.5–7.5 was obtained from a commercial nuclear pharmacy. The volumes injected were adjusted using 0.9% NaCl, USP (Hospira). Hynic-Annexin V kit preparation vials were supplied by the NIH National Cancer Institute. Hynic-Annexin V kit vials were stored at −80°C prior to use.

A Hynic-Annexin V vial containing 0.275 mg and containing a lyophilized stannous tricine buffer were thawed at room temperature (22°C) for 5 to 8 minutes. Approximately 30–50 mCi of ^99m^Tc (sodium pertechnetate) in 0.5 mL were added to the Hynic-Annexin V vial and gently swirled. Three mL of normal saline (Hospira) were added to the stannous tricine. After mixing the solution, 300 µL of the tin solution w withdrawn and added to the annexin solution. The solution was mixed and allowed to incubate at room temperature for 15 minutes.

The radionuclide incorporation yield was determined using a citrate-dextrose solution (Sigma). The radiolabeled compound (2–5 mL) was spotted on an iTLC chromatographic strip (Silica Gel Impregnated Glass Fiber Sheets, Varian). The iTLC strips were scanned on an AR2000 (Bioscan Inc.). The radioactivity at the origin (Rf = 0–4.25 cm), middle (Rf = 4.25–4.75 cm), and solvent front (Rf = 4.75–9 cm) were analyzed to determine the radiolabeling yield. based on incorporation yield, impurities and free/reduced-hydrolyzed ^99m^Tc radioactivities, respectively. The percent incorporation yields were calculated by taking the origin divided by the sum of the origin, middle and solvent times 100. Greater than 90% radiolabeling incorporation yield was required for acceptance. In addition, the middle section of the strip (impurities) had to contain ≤5% of the total radioactivity. The pH of the solution was measured to ensure it was in the specified range of 5.5 to 6.5. The final volume was adjusted according to injection volumes needed following quality assurance end-product testing.

### Radioimaging Studies

SPECT/CT studies were performed utilizing a NanoSPECT/CT® dedicated small-animal imaging system (Bioscan, Inc. Washington, DC). Imaging of a limited cohort of resveratrol- or saline-treated rats (n = 3/group) was performed at baseline, then again on days 7, 14, 21, 28, 35, and 42 post-monocrotaline injection. Thirty (30) minutes prior to imaging, rats were injected with ^201^Tl (540 µCi, mean activity) via tail vein to evaluate cardiac perfusion and morphology and ^99m^Tc-Annexin (608 µCi, mean activity) to assess heart and lung apoptosis. Thoracic imaging studies were performed on anesthetized rats (1.5%–2.0% isoflurane) using a temperature-controlled bed. A dual-isotope protocol was used to acquire ECG-gated and static ^201^Tl and ^99m^Tc-Annexin images simultaneously. A crosstalk removal procedure was used prior to image reconstruction to account for spillover of counts between the ^201^Tl and ^99m^Tc energy windows. Due to a larger proportion of LV to RV mass and correspondingly greater LV ^201^Tl signal, analysis of LV volume was performed to compute ejection fraction, stroke volume and myocardial perfusion polar maps (FlowQuant, University of Ottawa Heart Institute). An automated analysis routine was developed to identify heart and lung regions of interest (ROIs) from the ^201^Tl static reconstruction. These ROIs were applied to the ^99m^Tc-Annexin static reconstruction to assess heart and lung apoptosis. Qualitative perfusion mapping images and reported values for ^201^Tl and ^99m^Tc-Annexin were normalized to injected dose of each radionuclide.

### Hemodynamics

Rats were anesthetized with isoflurane and following a media-lateral incision, the right external jugular vein was exposed by blunt dissection. RSVP measurements were made via a fluid-filled pressure transducer (Becton Dickinson, DTXplus) where a heparinized (0.01%), saline filled Micro-Renathane catheter (0.050 OD X 0.040 ID) was placed in the right jugular vein. Catheter advancement preceded until strong positive-negative deflections were observed, indicating catheter placement in the right ventricle, then secured with 4-0 silk suture. Pressure data was collected for 1 minute using a conventional data acquisition system (AD Instruments). Following mid-line thoracotomy, rats were euthanized by exsanguination and visual confirmation of catheter placement in either right ventricle or PA was examined. In addition to RV systolic pressure, post hoc analysis of RV mean pressure, heart rate and +*dP*/*dt_max_* was performed by using PowerLab software suite 4/30 (AD Instruments).

### Statistics

All data were tested for normal distributions prior to analysis. ^201^Tl and ^99m^Tc-Annexin image generation/analysis as well as assessments of hemodynamics were performed in a blinded fashion. As RV hypertrophy developed in the MCT cohort, modest to moderate detection of the ^210^Tl signal in the RV became observable, thus unintentional un-blinding of the ^201^Tl analysis within experimental PAH cohort developed. Despite this longitudinal observation, assumed to be associated with the development of right ventricular hypertrophy, *post hoc* (off-line) analysis of ^201^Tl and ^99m^Tc-Annexin imaging was performed in a blinded fashion. Hemodynamic and organ weight data were assessed by Analysis of Variance (ANOVA) with Newman-Keuls Multiple Comparison Test to establish group differences (GraphPad Prism, v5.02). Time-course and cardiac cycle data derived from imaging was analyzed with a 2-way ANOVA with Bonferroni *post hoc* testing. Probability values less than 0.05 were considered significant.
